# A unique arsenic profile with unusual arsenic compounds discovered in the edible mushroom *Sparassis crispa*

**DOI:** 10.1007/s00216-025-06201-7

**Published:** 2025-11-21

**Authors:** Lorenz Steiner, Andrea Raab, Bassam Lajin, Jan Borovička, Julia Truschner, Walter Goessler

**Affiliations:** 1 Institute of Chemistry, Analytical Chemistry, University of Graz, Universitätsplatz 1, 8010 Graz, Austria; 2 Institute of Geology, Czech Academy of Sciences, Rozvojová 269, 16500 Prague 6, Czech Republic; 3 Nuclear Physics Institute, Czech Academy of Sciences, Hlavní 130, 25068 Husinec-Řež, Czech Republic

**Keywords:** Speciation, Trace elements, Biological samples, Ion chromatography/ion exchange, Mass spectrometry/ICP-MS

## Abstract

The edible and medicinal mushroom *Sparassis crispa* contains a diverse profile of arsenic species. We investigated the arsenic profile of extracts from four different fruiting bodies of *S. crispa* originating from Austria and Czechia. Besides the previously known arsenic species arsenocholine, trimethylarsine oxide, trimethyl(2-carboxyethyl)arsonium, arsenobetaine amide, and the tetramethylarsonium ion, we were able to identify the uncommon α-glycerophosphorylarsenocholine—previously only identified as a minor arsenic species in marine animals—as a major arsenic species. Furthermore, a novel arsenic compound, i.e., β-methyl arsenocholine, was identified as a naturally occurring compound in all investigated samples of *S. crispa*. We present methods for the preparation of synthetic α-glycerophosphorylarsenocholine and β-methyl arsenocholine, which were used to confirm their presence in *S. crispa*.

**Graphical Abstract:**

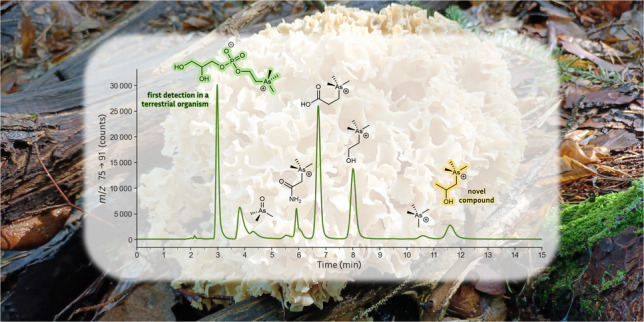

**Supplementary Information:**

The online version contains supplementary material available at 10.1007/s00216-025-06201-7.

## Introduction

**Fig. 1:**
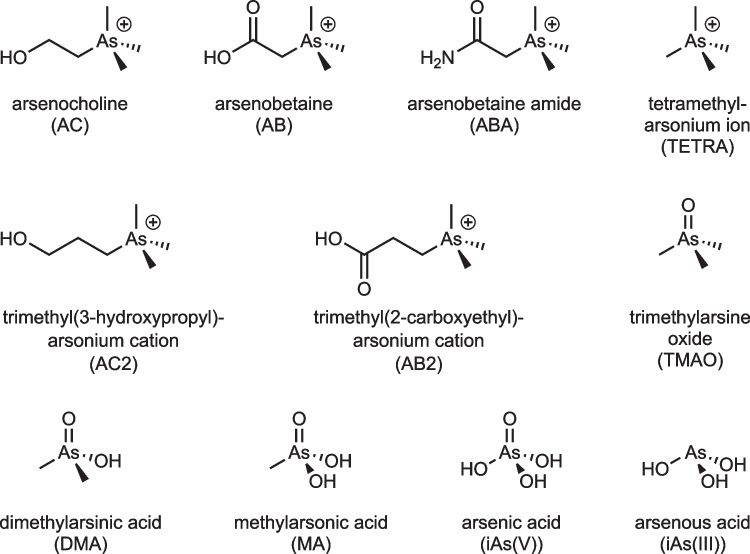
Organic arsenicals and inorganic arsenic, (iAs(III) and iAs(V)), commonly found in mushrooms

Fungi represent a major kingdom of life with a long evolutionary history tracing back to over 1 billion years ago [1]. Within the fungal kingdom, the Agaricomycetes is a large class encompassing fruiting body-forming Fungi. The function of the fruiting body (sporocarp, colloquially referred to as *mushroom*) is to produce spores, which are dispersed for reproduction. Even when growing at pristine sites, the fruiting bodies of many fungal species can accumulate arsenic for currently unknown reasons, usually in the form of one or more arsenic species (Fig. 1) [2, 3]. In most cases, the majority of arsenic is present as methylarsonic acid (MA), dimethylarsinic acid (DMA), arsenobetaine (AB), or inorganic iAs(III) or iAs(V) [2]. As evidenced by the structures in Fig. 1, the Me_3_As-moiety in organic arsenicals is a common motif that is present in many naturally occurring arsenic species, and a handful of homologues of AB have been found in mushrooms, including the relatively widespread arsenocholine (AC) [2]. Less common related species of AB include arsenobetaine amide (ABA) [4], homologues of AB and AC with an additional CH_2_ in the chain, i.e., trimethyl(2-carboxyethyl)arsonium (AB2) [5] and trimethyl(3-hydroxypropyl)arsonium (AC2) [6], as well as arsenocholine-*O*-sulfate, the sulfate ester of AC [7].

*Sparassis* (*Polyporales*, *Sparassidaceae*) is a genus of parasitic mushrooms growing throughout the northern hemisphere in European, Asian, and North American forests [8]. Many *Sparassis* species are consumed across different countries and are also valued as medicinal mushrooms [9–13], which is commonly attributed to their high β-glucan content [14]. The arsenic profile of the European species *Sparassis crispa* has been previously investigated using high-performance liquid chromatography (HPLC) coupled with inductively coupled plasma mass spectrometry (ICPMS), but at the time, the major arsenic compounds remained unknown [3]. Unlike most other mushrooms, where a single arsenic species (predominantly AB, DMA, MA, or inorganic As) makes up 50–100% of total arsenic [2], *S. crispa* contains a diverse and unique arsenic profile. Since *S. crispa* is a popular edible mushroom frequently consumed by humans, it is important to characterize its arsenic profile, as the toxicity of arsenic greatly varies with the species in question [15]. Inorganic arsenic, i.e., arsenite and arsenate, is considered highly toxic, with reported LD_50_ in the range of 15–48 mg As/kg [16]. While many organic arsenicals are generally considered barely toxic, this is certainly not a general rule, as DMA and MA have been shown to exhibit notable toxicity in human keratinocyte cultures [16, 17]. Furthermore, the toxicity of the lipophilic arsenolipids should not be underestimated, as they seem to have an influence on human neurons [18]. As mentioned above, an earlier study was not able to fully characterize the arsenic profile in the commonly eaten *S. crispa* [3]. The identification and quantification of arsenic species in this mushroom is thus the main goal of this study.

## Materials and methods

### Sample preparation

In this study, the fruiting bodies of multiple collections of *Sparassis crispa* from Austria and the Czech Republic were investigated. Austrian samples (labeled AT1, AT2, and AT3) were collected near Pöllau bei Hartberg (Styria, Austria) and stored in plastic bags during transportation. The fruiting bodies were freeze-dried (Christ, Osterode am Harz, Germany) and homogenized (ultra-centrifugal mill ZM200, 1 mm titanium sieve, Retsch GmbH, Haan, Germany). The Czech sample (CZ1) was collected at Mokrsko village in Central Bohemia, where As content in soil is naturally elevated [19] and was provided as a homogenized powder.

### Total arsenic determination

Triplicates of the sample powder (200 mg each) and a certified reference material (CRM) IPE-120 (250 mg, *Agaricus bisporus*, WEPAL, Wageningen, Netherlands) in 5 mL of HNO_3_ (p.a., ≥ 65%, Carl Roth, Karlsruhe, Germany, sub-boiled in-house) were subjected to microwave-assisted digestion (Ultraclave IV, MLS GmbH, Leutkirch, Germany; temperature ramp up to 250 °C, pressure up to around 100 bar). Due to the low amount available, a triplicate measurement of the sample from Czechia was not possible; thus, the total As concentration in CZ1 is based on only a single measurement. To the digests, 500 µL of concentrated HCl were added and diluted to a total volume of 50 mL with ultrapure water (18.2 MΩ × cm, Merck Millipore, Bedford, USA). Arsenic was determined by ICPMS (7900, Agilent Technologies, Waldbronn, Germany) or ICPMS/MS (8900, Agilent Technologies, Waldbronn, Germany). The quality of the analysis was confirmed by the aforementioned reference material CRM IPE-120 and the standard reference material SRM® 1643f (Trace elements in water, NIST, Gaithersburg, USA), as well as blanks consisting of ultrapure water and nitric acid added to the autoclave during digestion. The trueness of the measurement was confirmed by comparing the measured to expected arsenic concentration. The deviation was calculated by (*c*_*measured*_*–c*_*expect*_)/*c*_*expected*_ × 100%, arriving at + 1% relative deviation for CRM IPE-120, and –11% for SRM® 1643f, respectively.

### Sample extraction

Arsenic species were extracted by weighing homogenized mushroom samples (200 mg for AT1–AT3; 10 mg for CZ1) in a 15 mL polypropylene tube and adding ultrapure water (4 mL for AT1–AT3; 2 mL for CZ1). The limited amount of sample CZ1 did not allow for use of more material. The tubes were placed in an ultrasonic bath at ambient temperature for 30 min; the phases were separated by centrifugation (15 min at 10,000 g), and the supernatant was heated to 90 °C for 30 min. Following this heating step, additional solids precipitated from solution, which were removed by centrifugation (20 min at 21,000 g) and filtration of the supernatant through a 0.22 µm Nylon syringe filter to give a clear extract. Unheated extracts would become cloudy within 1–2 days of preparation due to the formation of a fine precipitate, leading to potential problems when injecting samples into the HPLC system. Furthermore, the addition of H_2_O_2_ to unheated extracts led to vigorous gas development. This was likely due to enzymatic activity, as no gas development was observed when adding H_2_O_2_ to heated extracts. Preliminary experiments revealed that heating the supernatant to 90 °C did not have an apparent effect on the arsenic species, and recorded chromatograms were indistinguishable before and after heating. To study the levels of inorganic arsenic, oxidation of potentially present inorganic As^3+^ to As^5+^ was achieved by adding 30% H_2_O_2_ to a heated extract (1 + 9) and heating the sample to 60 °C for 30 min. To determine the extraction efficiency, 200 µL of the extracts was added to 5 mL of 10% nitric acid and 1% HCl in 5 mL of ultrapure water. These samples were measured alongside the microwave-assisted acid-digested samples when determining the total arsenic content. An extraction efficiency of 98 ± 12% was determined.

### Arsenic speciation

Arsenic species were separated on an Agilent 1290 UHPLC system (Agilent Technologies, Waldbronn, Germany) comprising a binary pump, an autosampler, and a heated column compartment. Chromatographic conditions for cation-exchange and anion-exchange chromatography are summarized in Table 1. For the preparation of the mobile phases, ammonium formate (≥ 99.95% trace metal basis, Sigma-Aldrich) or diammonium hydrogen phosphate (puriss p.a., ACS reagent, ≥ 99.0%, Sigma-Aldrich) was used. The pH of the former was adjusted with formic acid (puriss. p.a. ≥ 98%, Sigma-Aldrich), while the latter was adjusted with 25% aqueous ammonia solution (ultra pure, Chem-Lab). High-resolution mass spectra (HRMS) were obtained from an Agilent ESI-TOF (electrospray ionization time of flight) with a capillary voltage v_c_ = 4000 V, and a nozzle voltage v_n_ = 2000 V. Chromatographic conditions are summarized in Table 1 under the cation-exchange header.

**Table 1:** Chromatographic conditions employed for the separation of anionic and cationic arsenic species

Parameters	Anion-exchange	Cation-exchange
Stationary phase	Hamilton PRP-X100	Zorbax 300-SCX
Length × diameter (mm)	150 × 4.6	150 × 4.6
Particle size (µm)	5	5
Mobile phase	20 mM (NH_4_)_2_HPO_4_, pH 6.0	30 mM NH_4_HCO_2_, pH 2.3
Flow (mL/min)	1.0	1.5
Temperature (°C)	40	40

### Synthesis of arsenic standards

#### General considerations

All air-sensitive reactions were carried out under nitrogen using standard Schlenk techniques. AsCl_3_ was synthesized from As_2_O_3_ and SOCl_2_ according to the literature [20], and then further converted to AsMe_3_ in a Grignard-type reaction according to the literature [21]. Arsenocholine, 1-bromo-2-propanol, and (2,2-dimethyl-1,3-dioxolane-4)methanol were synthesized in-house.

#### Synthesis of trimethyl(2-hydroxypropyl)-arsonium

The novel trimethyl(2-hydroxypropyl)-arsonium cation, or β-methyl arsenocholine (MeAC) as of established trivial naming schemes [22], was synthesized analogous to other arsenocholine homologues by combining trimethylarsine with an equimolar amount of 1-bromo-2-propanol under inert conditions [4, 23]. A detailed description of the procedure is given in the supplementary information. In short, the nucleophilic attack of bromide at propylene oxide gave a mixture of 1-bromo-2-propanol and 2-bromo-propanol in an 80/20 ratio. Addition of trimethylarsine to this mixture gave only a single product, albeit in a low yield of 17%. This is likely due to a combination of the higher proportional presence of 1-bromo-2-propanol and its less sterically demanding electrophilic carbon, which may be attacked more readily.

Found *m/z* for C_6_H_16_AsO: 179.0411; isotope/mass accuracy score: Δppm –0.51 determined by LC-ESI-HRMS.

#### Synthesis of α-GPAC

**Scheme 1:**
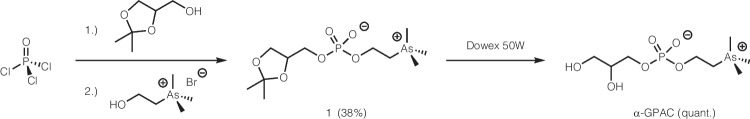
Synthesis of acetonide-protected α-GPAC (**1**), and subsequent deprotection under aqueous, acidic conditions. a: 1.) (2,2-Dimethyl-1,3-dioxolan-4-yl)methanol (1.0 equiv.), NEt_3_ (2 equiv.), DCM, 0 °C, 1 h; 2.) arsenocholine (1.0 equiv.), pyridine, 0 °C to rt, 16 h. b) Dowex 50 W Hydrogen form

The phosphate diester α-glycerophosphorylarsenocholine (α-GPAC) was prepared by adjusting a literature procedure for the synthesis of α-glycerophosphorylcholine [24]. As depicted in Scheme 1, to a solution of phosphoryl chloride in methylene chloride, an equimolar amount of 2,2-dimethyl-1,3-dioxolane-4-methanol and excess NEt_3_ in methylene chloride, followed by an equimolar amount of arsenocholine in pyridine, were added, and the reaction was allowed to run to completion. The reaction mixture was subsequently quenched with water, forming the acetonide-protected α-GPAC (**1**). The crude, aqueous reaction mixture was subjected to HPLC–ICPMS, which revealed only arsenocholine (25%) and **1** (75%) as the two major arsenic species. Arsenocholine was removed by passing the product solution through Dowex 50 W Hydrogen form cation-exchange resin. The low pH of the resin resulted in the deprotection of the acetonide and the formation of α-GPAC from **1**, as evidenced by HPLC–ICPMS. Formation of the product was confirmed by HPLC-ESI-HRMS, where we measured *m/z* for C_8_H_20_AsO_6_P: 319.0286; isotope/mass accuracy score: Δppm –0.06. Attempted isolation of solid α-GPAC by lyophilization was unsuccessful, as a sticky, brown residue was obtained each time. While the only arsenic species in the product solution prior to lyophilization was α-GPAC (> 98%), notable amounts of arsenocholine and other unidentified arsenic species were detected in the lyophilizate. The final yield of 38% of α-GPAC was therefore calculated from the total As concentration in solution after passing it through the cation-exchange resin to remove any cationic arsenic-containing impurities. Subjecting synthetic α-GPAC to alkaline conditions (0.5% NaOH) at 60 °C for 15 min resulted in its decomposition to the trimethyl-vinyl arsonium ion (TMVA), AC, AB, and inorganic arsenic.

## Results and discussion

### Total arsenic content in Sparassis crispa

The total arsenic content in the fruiting bodies of *S. crispa* was 0.69 ± 0.02 (AT1), 0.45 ± 0.01 (AT2), and 1.8 ± 0.2 (AT3) mg/kg dry mass in the Austrian samples, while the Czech sample collected from the arsenic-rich sample site was measured at 7.2 mg/kg dry mass. Uncertainties are given as the standard deviation from the triplicate measurements. The measurements are well above the limit of detection of 0.002 mg/kg dry mass.

### Anion-exchange chromatography

**Fig. 2:**
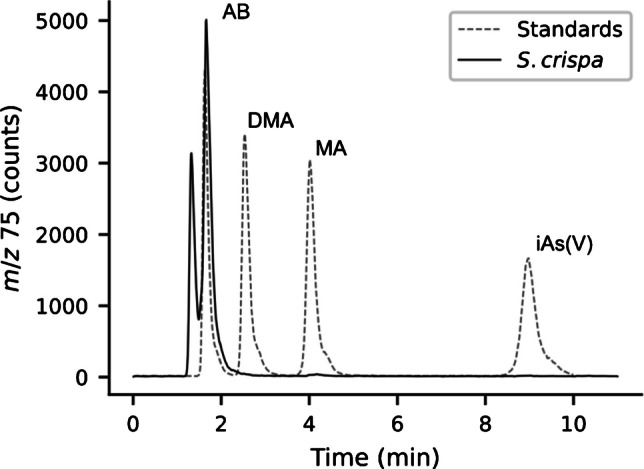
Anion-exchange chromatography of an aqueous extract of *Sparassis crispa* (solid line) and a mixture of As-standards (dashed line) arsenobetaine (AB), dimethylarsinic acid (DMA), methylarsonic acid (MA), and arsenate (iAs(V), 50 µg/L each). Conditions are summarized in Table 1. Note that the presence of AB cannot be definitively investigated using these conditions due to poor retention under anion-exchange conditions and therefore other chromatographic conditions were used (Fig. 3)

Arsenic species in oxidized and unoxidized aqueous extracts of *S. crispa* were then investigated employing HPLC–ICPMS. Spiking the extracts with standards of known arsenic compounds allowed for the identification of arsenic species. Figure 2 depicts the chromatogram obtained from anion-exchange chromatography on a Hamilton™ PRP-X100 column. Only two peaks with retention times close to or at the void time are resolved, indicating the absence of negatively charged arsenic species, e.g., DMA, MA, or arsenate, altogether. Neither the unoxidized nor oxidized extract contained detectable amounts of arsenate, suggesting the absence of inorganic arsenic in *S. crispa*.

### Cation-exchange chromatography

**Fig. 3:**
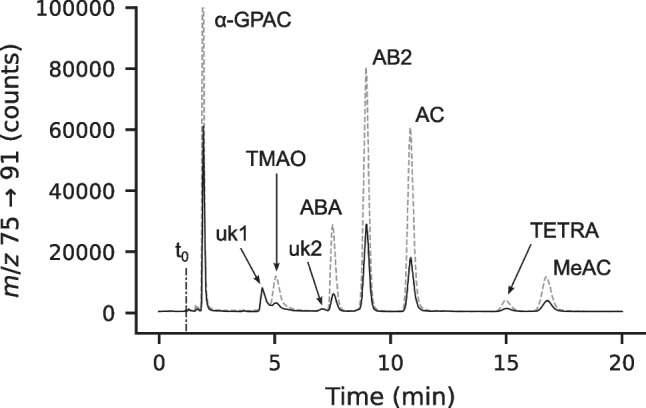
Superimposed cation-exchange chromatograms of an aqueous extract of *Sparassis crispa* (solid line), and the same extract spiked standards (dashed line) of α-GPAC, TMAO, ABA, AB2, AC, TETRA, and MeAC. The delay volume is indicated by dash-dotted line marked t_0_. Conditions are summarized in Table 1. Two unknown compounds (uk1 and uk2) remained unidentified

Separation of arsenic species was achieved on a Zorbax 300-SCX cation-exchange column. Despite the variation in total arsenic between fruiting bodies—which is likely attributed to varying arsenic levels between the collection sites—the arsenic profiles were constant. A column recovery of 95 ± 3% was determined by comparing the integral over the whole chromatogram with the integral of an injection where the column was bypassed completely. The uncertainty represents the standard deviation obtained from injecting each sample once (i.e., *n* = 4). Oxidation of the extracts with H_2_O_2_, as described above, did not have a noticeable effect on the arsenic species found in the extract, and oxidized and unoxidized extracts resulted in identical chromatograms. As evidenced by the chromatogram in Fig. 3, at least nine distinct arsenic species were found in the aqueous extracts of *S. crispa*, which is an unusually high number of arsenic compounds detected in a single species, indicating exceptional biochemical diversity for arsenic in this mushroom. By spiking the extracts with arsenic standards that were available to us, we could readily identify arsenocholine (AC), trimethyl(2-carboxyethyl)arsonium (AB2), arsenobetaine amide (ABA), trimethylarsine oxide (TMAO), and the tetramethylarsonium ion (TETRA).

The absence of significant amounts of arsenobetaine (AB) was confirmed by spiking extracts with standards (see Fig. S5 in the supporting information) and is striking, since the structurally related AC and AB2 constitute major arsenic species, and ABA was detected in notable amounts as well. Our findings stand in contrast to a previous investigation on the arsenic species in higher fungi, where two samples of *S. crispa* were investigated [3]. While AC was identified as the major species in both – 66% and 45%, respectively – the overall arsenic profile varied significantly, one containing 31% of AB, while only traces were found in the other. This discrepancy is notable, as we arguably found a characteristic arsenic profile across our samples, and all identified compounds were present in each investigated sample. Only a single chromatogram of an extract of *S. crispa* is printed in the earlier publication, which makes a thorough comparison not feasible. Nonetheless, the absence of AB in the samples measured by us is notable, as AB is among the most common arsenic species found in mushrooms [2], adding to the unusualness of its absence in *S. crispa *(Table 2).

**Table 2:** Arsenic species found in aqueous extracts of four different samples of *Sparassis crispa* identified by cation-exchange chromatography. Retention times match the chromatogram shown in Fig. 3. Reported values from a single measurement of each sample

Arsenic species	rt (min)	Relative peak area (%)			
		AT1	AT2	AT3	CZ1
Unretained As	< 1.8	1.5	0.7	1.3	0.5
α-GPAC	2.0	12	22	10	23
uk1	4.5	11	7.9	5.3	12
TMAO	5.0	7.7	4.9	9.8	5.4
ABA	7.5	7.7	5.8	7.5	5.9
AB2	9.0	33	30	34	35
AC	10.8	16	20	24	14
TETRA	15.0	1.6	1.8	3.1	1.0
MeAC	16.7	7.9	6.5	4.4	1.1

### α-Glycerophosphorylarsenocholine

The major arsenic species eluting at 2.0 min in the cation-exchange chromatogram (Fig. 3) could initially not be matched with any arsenic standard available in our library. Thus, we attempted to identify the arsenic species by LC-ESI-HRMS and found a matching *m/z* for α-glycerophosphorylarsenocholine (α-GPAC). However, due to the zwitterionic nature of α-GPAC and the coelution of a plethora of other compounds, the signal-to-noise ratio was unsatisfactory. Ultimately, the presence of α-GPAC was confirmed by spiking the extracts with synthetic α-GPAC prepared in-house as described in detail in the supporting information.

The detection of α-GPAC in *S. crispa* is remarkable for multiple reasons. This not only marks the first instance of α-GPAC being detected in a terrestrial organism, but it has never been detected as a major arsenic species. To our knowledge, it is only mentioned twice in the literature in association with marine organisms. It was found occurring naturally in the anemone *Anemonia sulcata* [25], and was also detected in yellow-eye mullet (*Aldrichetta forsteri*) tissue after feeding them large quantities of arsenocholine [26]. In both cases, it constituted only a minor arsenic species accounting for < 3% of total arsenic. It is thus striking that α-GPAC constitutes a major arsenic compound in *S. crispa* accounting for ca. 10–20% of total arsenic. Its nitrogen analogue, α-glycerophosphorylcholine, is very common in biological systems, making up the building block for phospholipids and a wide range of choline derivatives.

### β-methyl arsenocholine

**Fig. 4:**
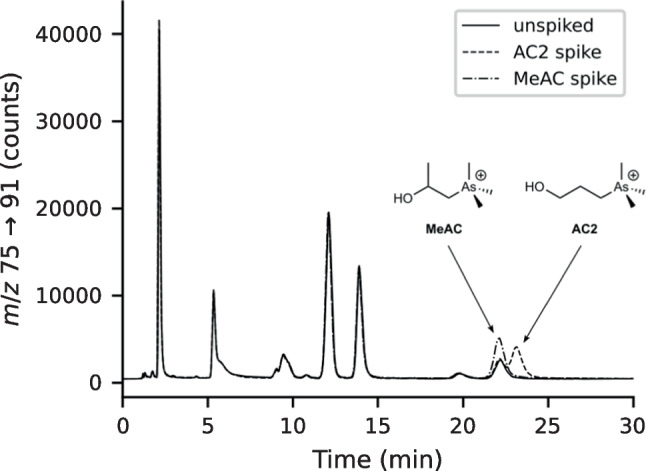
Cation-exchange chromatogram of an extract of *Sparassis crispa* (solid line) superimposed with chromatograms of the same extract spiked with AC2 (dashed line) or MeAC (dash-dotted line) with the column compartment kept at 20 °C

While optimizing column compartment temperature in cation-exchange chromatography, we noticed the retention time of the last eluting peak in the *S. crispa* extract, initially suspected to be trimethyl(3-hydroxypropyl)arsonium (AC2), did not match the retention time of the AC2-standard in our library. However, investigating the peak using LC-ESI-HRMS, a *m/z* matching that expected for AC2 was found. This prompted us to consider the structural isomer β-methyl arsenocholine (MeAC), which was synthesized as described above. We found that AC2 and MeAC coeluted at 40 °C, but a slight shift in retention time was noticeable at 30 °C, which was further emphasized at 20 °C (Fig. 4). The decrease in temperature, however, resulted in chromatograms of worse quality at lower retention times, and we could not arrive at conditions at which we were satisfied with both the separation of early eluting species and the separation of AC2 and MeAC, respectively. Nonetheless, the identification of MeAC as a novel naturally occurring arsenic species is surprising. Its chemical similarity to AC2 may be the reason why it has avoided identification thus far and may actually be a more widespread compound in mushrooms and animals altogether. This begs the question of whether or not previous reports on AC2 in mushrooms [6] are questionable and whether these samples should be reinvestigated, if only to confirm the uniqueness of the arsenic profile of *S. crispa*. The synthesis of the nitrogen analogues, i.e., homocholine and its structural isomers, has collectively been reported as early as 1932, and publications about the preparation of individual homologues may well predate it [22]. It is therefore surprising that the detection of its arsenic-containing homologue has not yet been reported in the literature.

### Decomposition of α-GPAC to TMVA

**Scheme 2:**
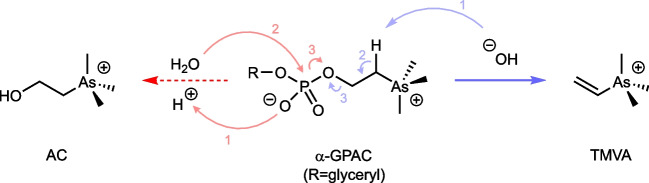
Proposed decomposition pathways of α-GPAC indicated by numbered arrow pushing steps under acidic conditions (left pathway highlighted in red, not observed) or basic conditions (right pathway highlighted in blue, observed)

While the synthesis of a standard of α-GPAC was ongoing, we wanted to attempt the derivatization of α-GPAC in the extract to a more readily ionized product. We subjected extracts of *S. crispa* to acidic and basic conditions, respectively, expecting either the hydrolysis of the phosphate ester in the former and the elimination of glycerophosphate in the latter case, as depicted in Scheme 2. Thus, extracts containing 50 mg freeze-dried *S. crispa* power/mL were prepared, adding solutions of either HCl or NaOH (5 weight% each) in a ratio of 9 + 1 (*S. crispa* extract + acid or base). The acidified/basified extracts were heated to 90 °C for 30 min before recording the chromatogram. While the addition of HCl resulted in chromatograms indistinguishable from the native extract, notable changes occurred after the addition of NaOH. The newly formed peak was identified as the trimethyl-vinyl-arsonium cation (TMVA) by LC-ESI-HRMS (measured *m/z* for C_5_H_12_As: 147.0171; isotope/mass accuracy score: Δppm 0.73). The formation of TMVA was then more thoroughly investigated, and extracts were subjected to a series of varying temperatures for 15 min ranging from 40–80 °C (Fig. S6 in the supporting information). This way, the gradual degradation of α-GPAC could be followed, as the intensity of the peak corresponding to TMVA increased (Fig. S6 in the supplementary information). The same decomposition was observed when subjecting synthetic α-GPAC to alkaline conditions. TMVA does not constitute a naturally occurring arsenic species, yet reporting its identification seems necessary, as no toxicological data for TMVA exist yet. While high pH environments are unlikely during cooking this edible mushroom, the possibility of its formation during prolonged heating is still conceivable.

## Conclusion and outlook

Unlike most other mushrooms, the arsenic profile of *S. crispa* is surprisingly diverse, containing at least ten distinct arsenicals, many of which are derivatives of arsenocholine. It contains the highest reported concentration of the sparingly explored α-glycerophosphorylarsenocholine, the only recently discovered arsenobetaine amide, and the novel arsenic species β-methyl arsenocholine. The latter elutes at a very similar retention time as unbranched homoarsenocholine. It is therefore required to investigate previously published conditions for their capability to separate AC2 from MeAC, and, if these fail to give satisfactory resolution of the two, optimize the conditions used for cation-exchange chromatography with these isomers in mind. In light of the high concentrations of arsenocholine and trimethyl(2-carboxyethyl)arsonium, the complete absence of arsenobetaine is striking. It is furthermore unclear whether *S. crispa* possesses distinct metabolisms for its nitrogen- and arsenic-containing choline derivatives, or if the incorporation of arsenic over nitrogen in the latter is accidental. A follow-up study investigating the absolute and relative (arseno)choline derivatives may certainly be insightful. Furthermore, the investigation of other *Sparassis* species or phylogenetically related fungal genera could provide knowledge of whether the diverse arsenic profile observed in this study is exclusive to *S. crispa*, the genus *Sparassis*, or if it is a more common feature in this group of fungi.

## Supplementary Information

Below is the link to the electronic supplementary material.

Supplementary Material 1 (DOCX 8.21 MB)

## Data Availability

Raw data for ICPMS and LC-ESI-TOF measurements is available upon request from the authors.
