# Supplementary material for: A unique arsenic profile with unusual arsenic compounds discovered in the edible mushroom Sparassis crispa
Source: Anal Bioanal Chem. 2025 Nov 21;418(16):5151–8. doi: 10.1007/s00216-025-06201-7 (PMC13424249; doi:10.1007/s00216-025-06201-7)
Supplement: Supplementary file 1 — Supplementary Material 1 (DOCX 8.21 MB) [file 216_2025_6201_MOESM1_ESM.docx]

Supplementary Information

A unique arsenic profile with unusual arsenic compounds discovered in the edible mushroom *Sparassis crispa*

*Lorenz Steiner,*^1^*Andrea Raab,*^1^ *Bassam Lajin,*^1^ *Jan Borovička,*^2,3^ *Julia Truschner,*^1^ *Walter Goessler*^1^

^1^Institute of Chemistry, Analytical Chemistry, University of Graz, Universitätsplatz 1, 8010 Graz, Austria

^2^Czech Academy of Sciences, Institute of Geology, Rozvojová 269, 16500 Prague 6, Czech Republic

^3^Czech Academy of Sciences, Nuclear Physics Institute, Hlavní 130, 25068 Husinec-Řež, Czech Republic

Table of Contents

[1. Syntheses 3](#_Toc209781313)

[1.1. bromo-propyl alcohols 3](#_Toc209781314)

[1.2. β-methyl-arsenocholine 3](#_Toc209781315)

[1.3. α-glycerophsophorylarsenocholine (α-GPAC) 4](#_Toc209781316)

[2. Sample information 4](#_Toc209781317)

[3. Chromatograms 5](#_Toc209781318)

[3.1. Cation-exchange chromatograms of extracts of *S. crispa* 5](#_Toc209781319)

[*3.2.* Alkaline decomposition of α-GPAC in *S. crispa* 10](#_Toc209781320)

[4. ICPMS/MS setting 11](#_Toc209781321)

[5. ESI-HRMS 12](#_Toc209781322)

[5.1. ESI-MS/MS spectra β-methyl-arsenocholine (MeAC) 12](#_Toc209781323)

[5.2. ESI-MS/MS spectra of Trimethyl-vinyl-arsonium ion (TMVA) 13](#_Toc209781324)

[5.3. ESI-MS/MS spectra of α-glycerophosphorylarsenocholine (α-GPAC) 14](#_Toc209781325)

[6. NMR Spectra 15](#_Toc209781326)

[7. References 18](#_Toc209781327)

# Syntheses

## bromo-propyl alcohols

In a 250 mL round bottom flask, propylene oxide (1.21 g, 20.8 mmol) was dissolved in *tert*-butyl alcohol (80 mL) and tetrabutyl-ammonium bromide (20 g, 62 mmol) was added. Subsequently, ceric ammonium nitrate (2.8 g, 4.2 mmol) was added. The mixture was then refluxed for 2 h before adding water (150 mL) and extracting the product with diethyl ether (3 x 50 mL). The combined organic phases were dried over MgSO_4_, and all volatiles were removed under reduced pressure until about 15 mL remained in the flask. The product was isolated by vacuum distillation (p = 30–35 mbar, T_head_ = 58–65 °C) as a mixture of 2-bromo-1-propyl alcohol/1-bromo-2-propyl alcohol (20:80, estimated by ^1^H NMR spectroscopy). The final yield was 1.15 g (40%) of a colorless, oily liquid. Shifts in the ^1^H NMR spectrum match those reported in literature [1].

1-bromo-2-propyl alcohol

^1^H NMR (300 MHz, CDCl_3_) δ 4.23 (m, 1H, Me–(CBr**H**)–C) 3.75 (dd, *J* = 12.2, 4.3 Hz, 1H, C–C**H**_2_–C), 3.67 (dd, *J* = 12.2, 7.0 Hz, 1H, C–C**H**_2_–C), 1.69 (d, *J* = 6.7 Hz, 3H, –C**H**_3_) ppm.

2-bromo-1-propyl alcohol

^1^H NMR (300 MHz, CDCl_3_) δ 4.07 – 3.91 (m, 1H, –C**H**-(OH)(Me)), 3.50 (dd, *J* = 10.2, 3.6 Hz, 1H, C**H**_2_), 3.36 (dd, *J* = 10.2, 7.0 Hz, 1H, C**H**_2_), 1.29 (d, *J* = 6.3 Hz, 3H, C**H**_3_) ppm.

## β-methyl-arsenocholine

A 10 mL Schlenk flask was charged with AsMe_3_ (640 mg, 5.33 mmol) and 1-bromo-2-propanol (925 mg, 6.67 mmol). The flask was closed and the mixture was stirred at 45 °C for 16 h. The contents of the flask were still liquid after that time, so the flask was equipped with a reflux condenser and the contents were heated to 80 °C for 3 h. After letting the contents cool to room temperature, dry, deoxygenated diethyl ether (5 mL) was added to precipitate the product. The liquid phase was removed by syringe and the solids were washed with additional ether (2 x 3 mL) and dried *in vacuo* to afford 234 mg (17%) of β‑methyl-arsenocholine bromide as a colorless, hygroscopic powder.

C_6_H_16_AsO 179.0411; isotope / mass accuracy score: Δppm –0.51

^1^H NMR (300 MHz, DMSO) δ 5.41 (d, *J* = 5.1 Hz, 1H, OH), 4.36 – 3.91 (m, 1H, –C**H**(OHCH_3_)), 2.56 (dd, *J* = 13.4, 3.7 Hz, 1H, As–C**H**_2_–), 2.46 (dd, *J* = 10.5, 2.8 Hz, 1H, As–C**H**_2_–), 1.85 (s, 9H, As(C**H_3_**)_3_ ), 1.21 (d, *J* = 6.1 Hz, 3H, C–C**H**_3_) ppm.

^13^C NMR (75 MHz, DMSO) δ 62.2, 34.4, 24.9, 8.1 ppm.

## α-glycerophsophorylarsenocholine (α-GPAC)

Prepared by adjusting a literature procedure for α-glycerophsophorylcholine [2]. In a 25 mL Schlenk flask kept at 0 °C in an ice bath, POCl_3_ (100 µL, 1.07 mmol), was dissolved in dry, deoxygenated methylene chloride (3 mL). 2,2-dimethyl-1,3-dioxolane-4-methanol (150 mg, 1.13 mmol) and triethylamine (150 µL, 0.36 mmol) in methylene chloride (2 mL) were added and the mixture was stirred 1 h. A suspension of arsenocholine (275 mg, 0.26 mmol) in pyridine (1 mL) was added in one portion, the ice bath was removed and the mixture was stirred for 16 h. Ultrapure water (5 mL) was then added and the mixture was stirred vigorously for 2 h before separating the phases. The organic phase was discarded and the aqueous phase was carefully removed *in vacuo*. Acetone (5 mL) was added to the sticky residue and the mixture was stirred vigorously for 30 min before separating the phases. The supernatant was stored at –20 °C for several days, while the sticky, brown residue was dried *in vacuo*. The residue was then dissolved in ultrapure water (3 mL) and passed through a Dowex 50W H^+^-form cation exchange resin to remove any cationic impurities. The low pH of the resin also removed the acetonide protecting group. The isolation by lyophilization was repeatedly unsuccessful and resulted in the decomposition of the product. Hence, the final yield was calculated by determination of the total As-content in the product solution by ICPMS. In the total volume of 25 mL, a concentration of 1.3 mg As/mL was found, corresponding to 38% yield of α-GPAC.

C_8_H_20_AsO_6_P 319.0286; isotope / mass accuracy score: Δppm –0.06

# Sample information

Nine fruiting bodies of *Sparassis crispa* were collected near Pöllau (8225 Pöllau, Austria, Styria). Within a sampling area spanning approximately 5 km across, several fruiting bodies of *S. crispa* have been found, collected and labelled by roman numerals, i.e., *S. crispa* I–IX, each corresponding to an individual sampling site within the estimated 5 km. After freeze-drying, three fruiting bodies were picked at random and labeled ***S. crispa* AT1**, ***S. crispa* AT2**, and ***S. crispa* AT3**. Additionally, a pre-processed sample from the Czech Republic from an auriferous area naturally rich in topsoil arsenic content (***S. crispa* CZ1**) was used.

# Chromatograms

## Cation-exchange chromatograms of extracts of *S. crispa*


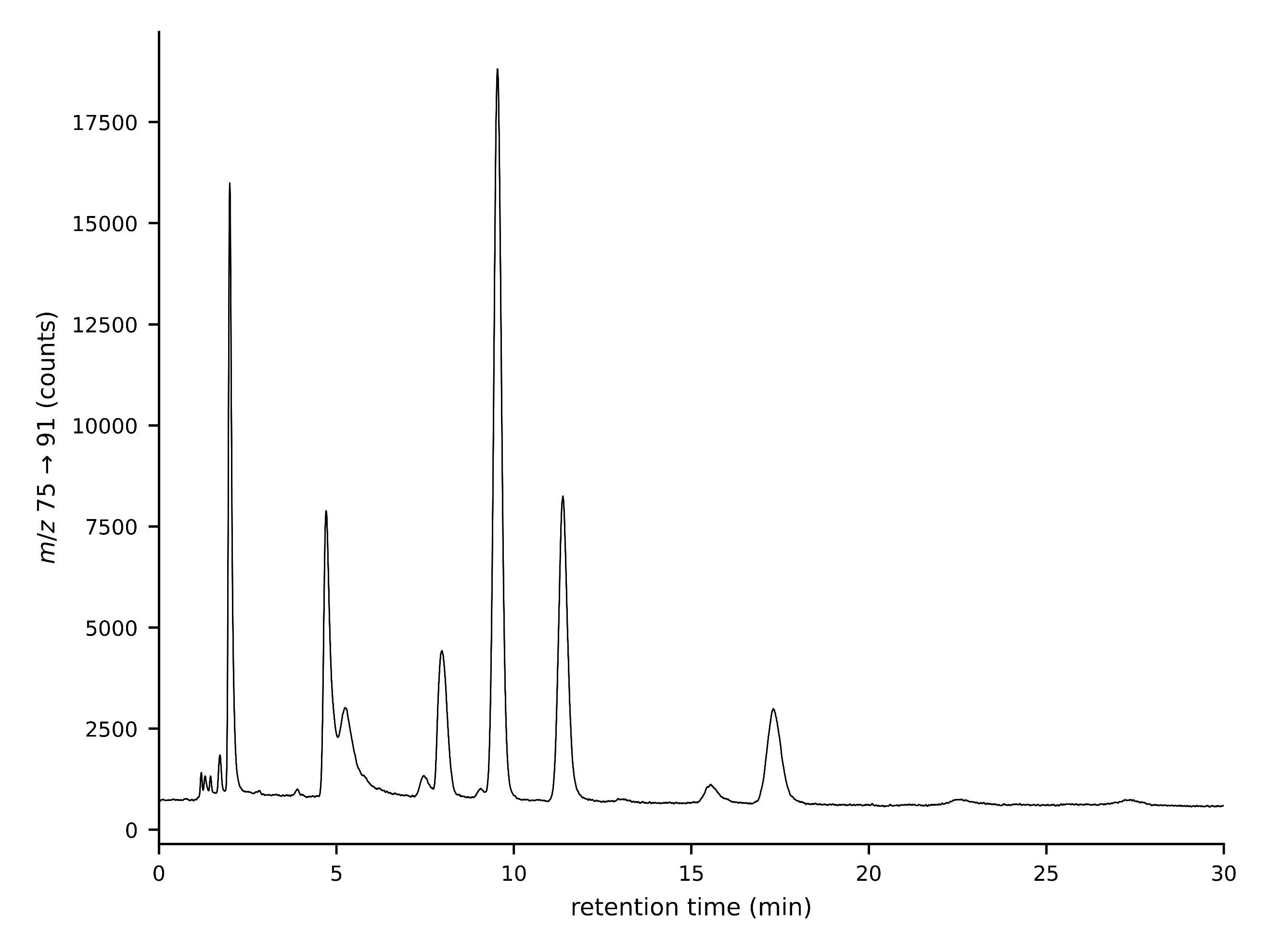


TETRA

α-GPAC

TMAO

ABA

AB2

AC

MeAC

Fig. S1 Cation-exchange chromatogram of an extract of *S. crispa* AT1


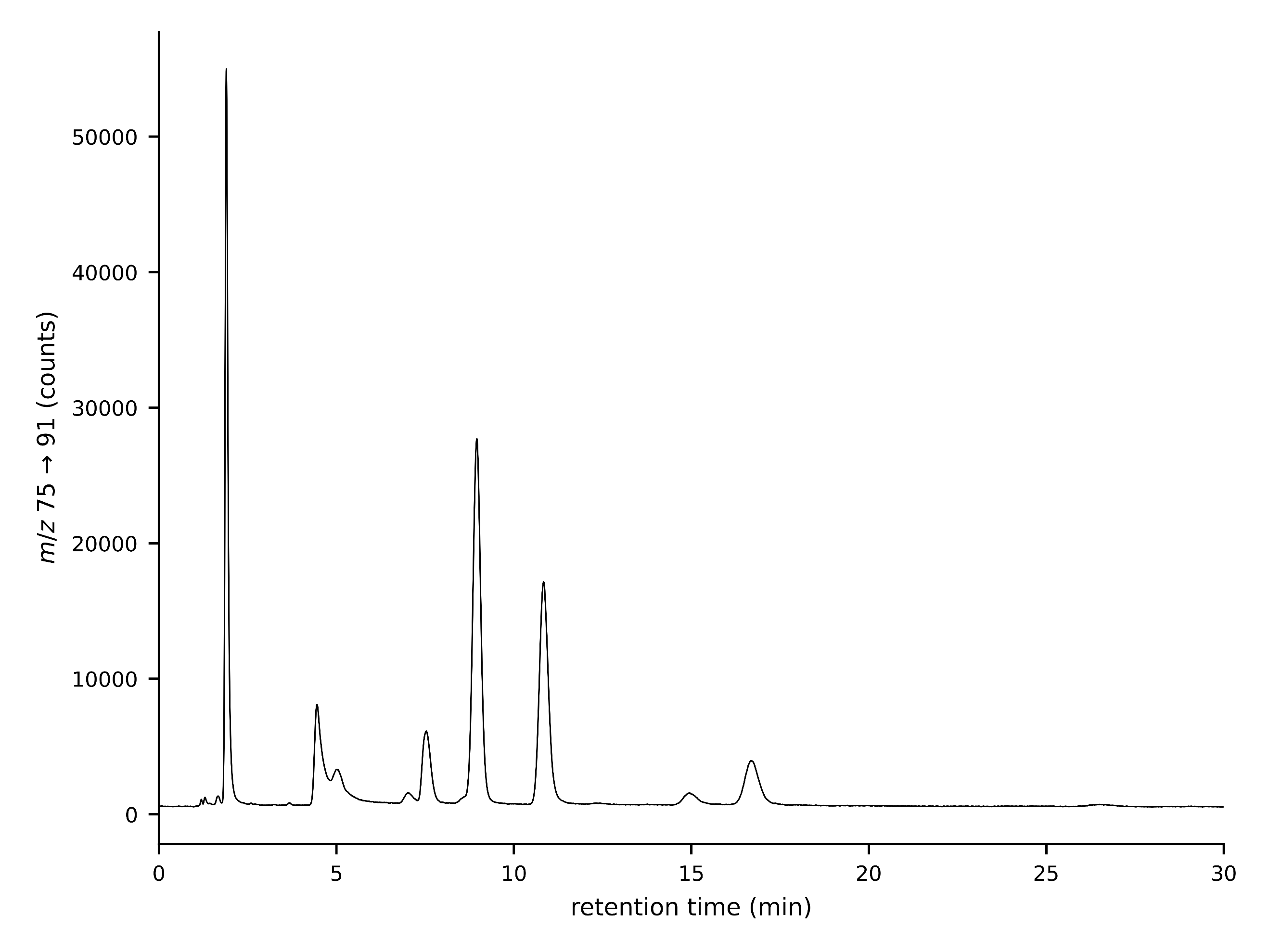


α-GPAC

TETRA

MeAC

AB2

AC

ABA

TMAO

Fig. S2 Cation-exchange chromatogram of an extract of *S. crispa* AT2


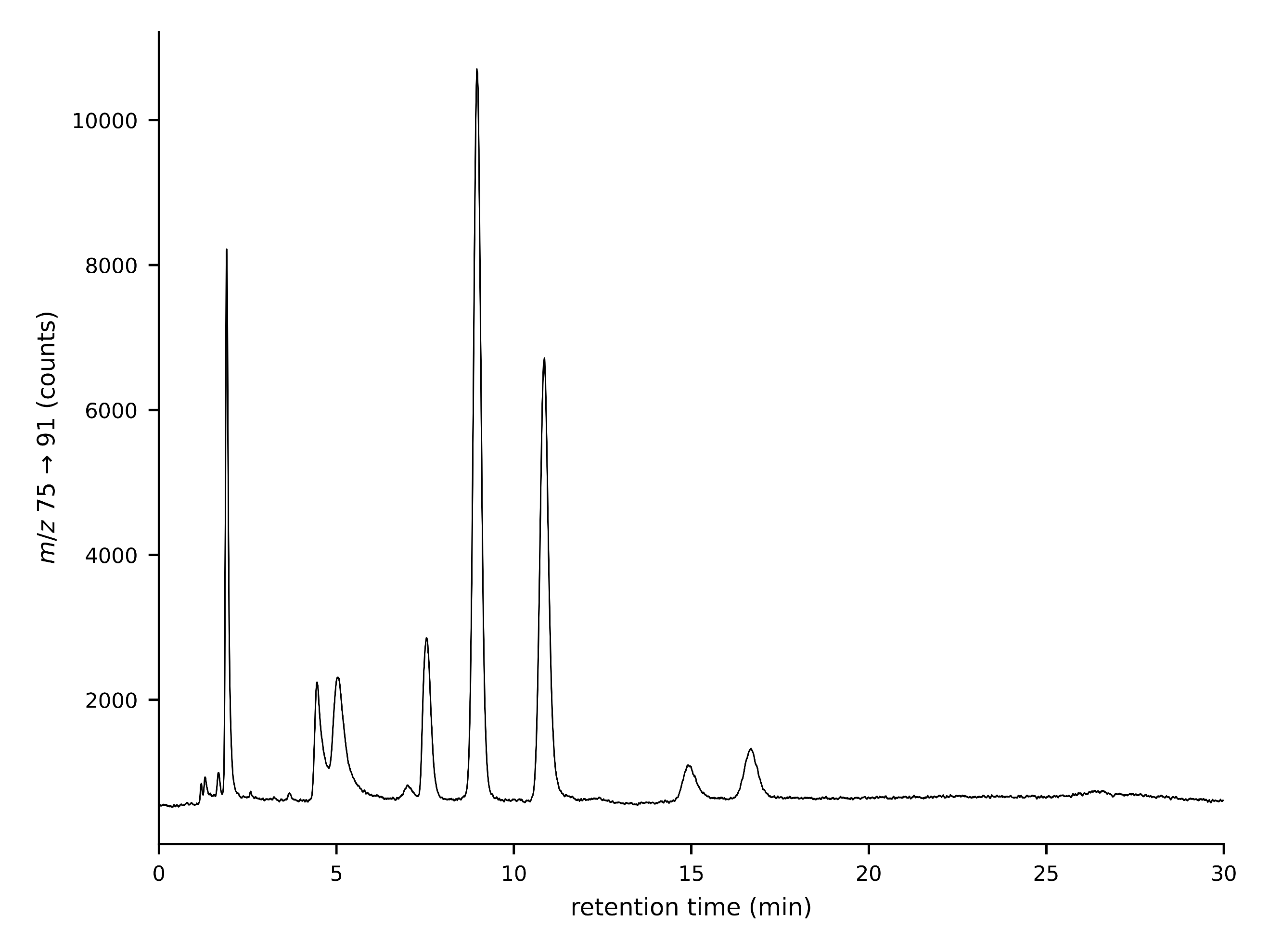


α-GPAC

MeAC

TETRA

AC

AB2

ABA

TMAO

Fig. S3 Cation-exchange chromatogram of an extract of *S. crispa* AT3


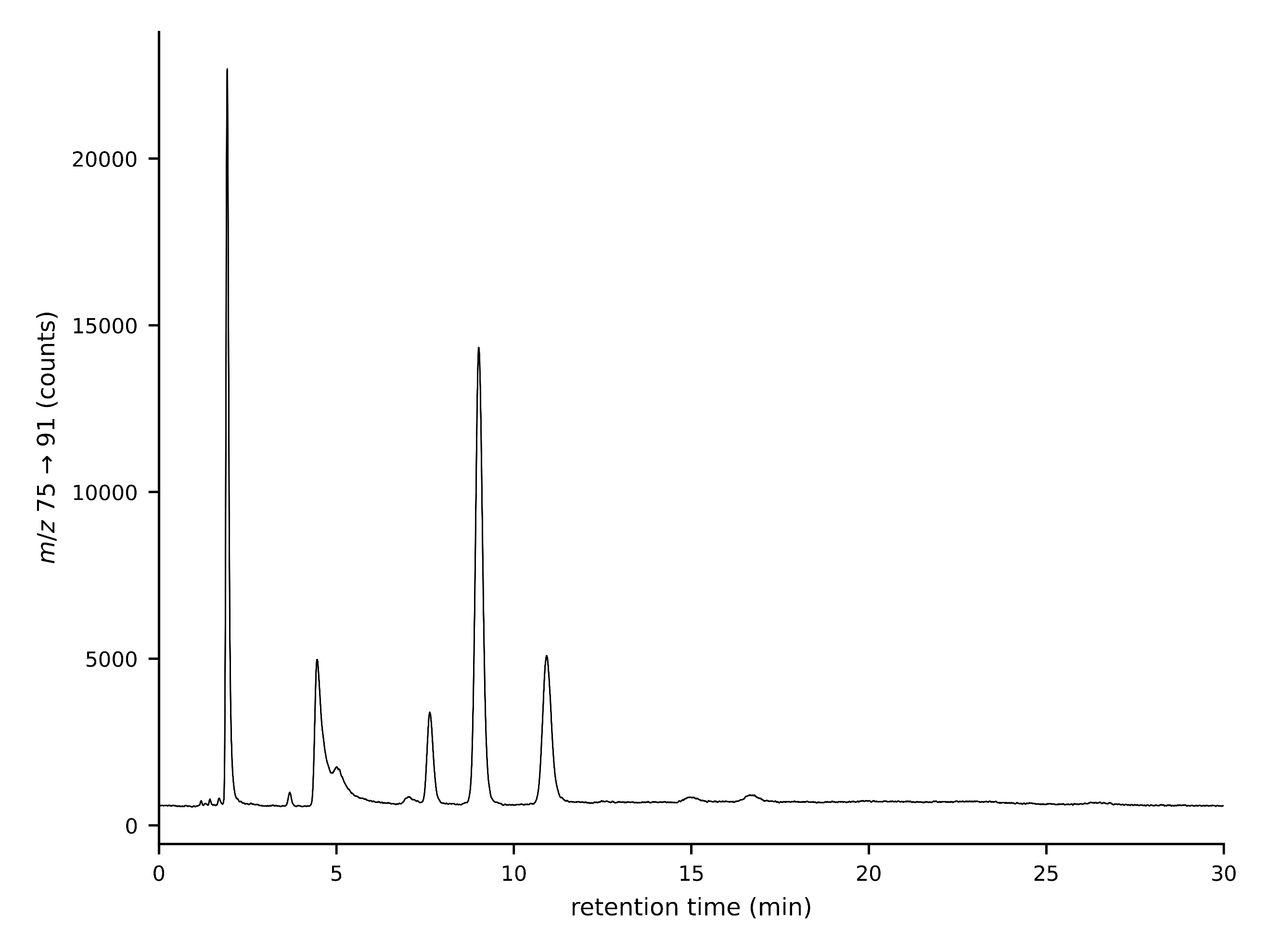


TMAO

α-GPAC

ABA

AB2

AC

TETRA

MeAC

Fig. S4 Cation-exchange chromatogram of an extract of *S. crispa* CZ1


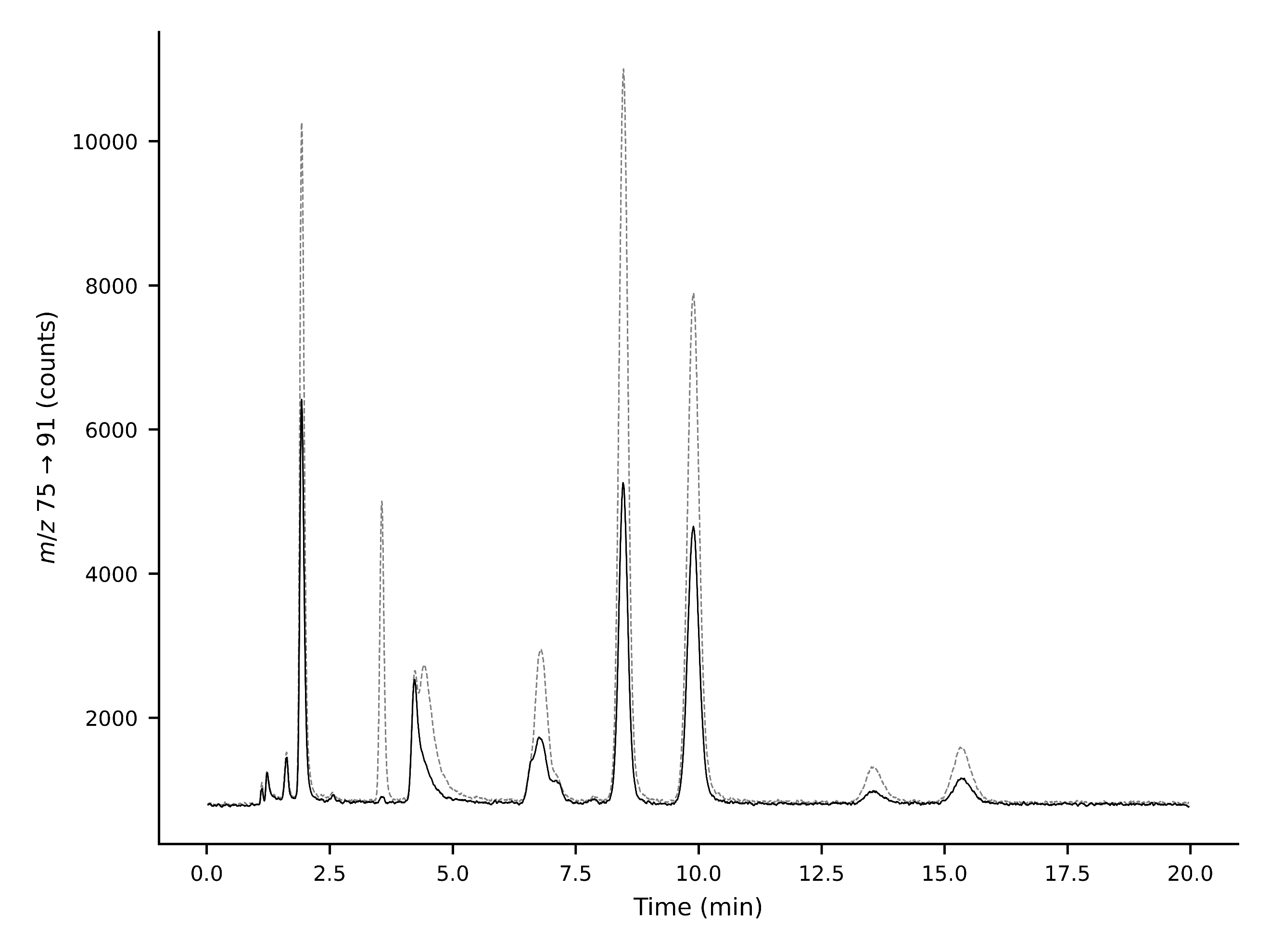


ABA

AC

MeAC

TMAO

AB

α-GPAC

AB2

TETRA

Fig. S5 Superimposed chromatograms of an aqueous extract of AT2 (black solid line) and the same extract spiked (gray dashed line) with a mix of eight arsenic standards (α-GPAC, AB, TMAO, ABA, AB2, AC, TETRA, and *i*-AC2 in order of ascending retention time)

## Alkaline decomposition of α-GPAC in *S. crispa*


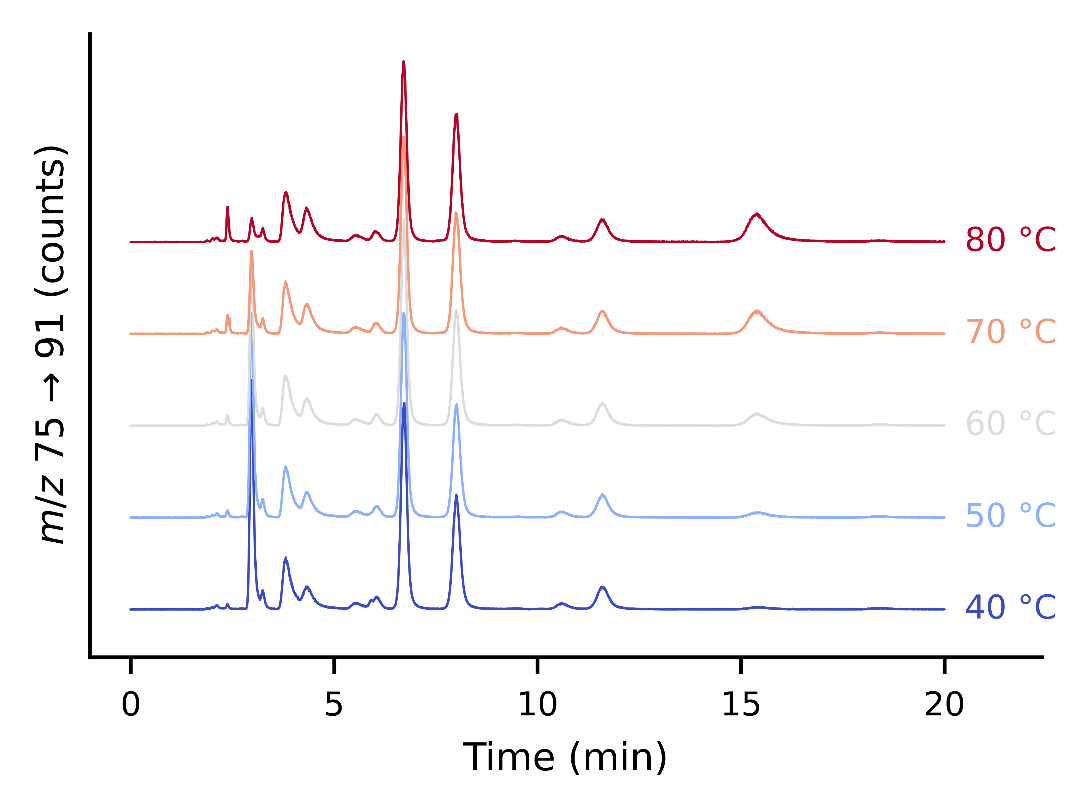


α-GPAC

TMVA

Fig. S6 Stacked chromatograms of an alkaline extract of *S. crispa* heated to 40–80 °C for 15 min showing the gradual degradation of α-GPAC to TMVA. Conditions slightly differ from the ones used for chromatograms shown in Fig S1–S4. A more degraded stationary phase (Zorbax 300-SCX (150×4.6 mm, 5 µm)) was used, resulting in lower overall retention times. Mobile phase: NH_4_HCO_2_ (30 mM, pH 2.70, 1 mL/min, 30 °C)

# ICPMS/MS setting

Table S1 ICPMS/MS parameters used for the analysis of arsenic in the LC-ICPMS/MS experiments

| **Plasma parameter** | **value** |
| --- | --- |
| Nebulizer Gas | 0.75 L/min |
| Option gas (1% CO_2_ in Ar) | 15% |
| Makeup Gas | 0.19 L/min |
| Plasma Gas | 15.0 L/min |
| Auxiliary Gas | 0.9 L/min |
| **Collision cell parameter** | **value** |
| He-flow | 1.0 L/min |
| 1% O_2_ in He (%) | 15% |

# ESI-HRMS

## ESI-MS/MS spectra β-methyl-arsenocholine (MeAC)

C_6_H_16_AsO 179.0411; isotope / mass accuracy score: 88; Δppm –0.51

## ESI-MS/MS spectra of Trimethyl-vinyl-arsonium ion (TMVA)

C_5_H_12_As 147.0171; isotope / mass accuracy score: 89; Δppm 0.73

## ESI-MS/MS spectra of α-glycerophosphorylarsenocholine (α-GPAC)

C_8_H_20_AsO_6_P 319.0286; isotope / mass accuracy score: 99; Δppm –0.06

# NMR Spectra

Fig. S7 ^1^H-NMR spectrum of a 80/20 mixture of 2-bromo-1-propyl alcohol and 1-bromo-2-propyl alcohol in CDCl_3_

TMAO

b

b

c

e

d

a

Fig. S8 ^1^H NMR-spectrum of β-methyl-arsenocholine bromide in DMSO-*d6*. Signals for one of the diastereotopic –CH_2_– are overlayed with the solvent signal at around 2.5 ppm

Fig. S9 ^13^C NMR-spectrum of β-methyl-arsenocholine bromide in DMSO-*d6*

References

1. Mirosław Soroka, Waldemar Goldeman, Piotr Maysa, Monika Stochaj. Comments on a Conversion of Epoxides to Halohydrins with Elemental Halogen Catalyzed by Phenylhydrazine: Tandem Electrophilic Halogenation of Aromatic Compounds and Epoxide Ring Opening to Halohydrins. Synthesis. 2003; https://doi.org/10.1055/s-2003-42424

2. Park J-M, Castro KA de, Ahn H-S, Rhee H-J. Facile Syntheses of L-α-Glycerophosphorylcholine. Bull Korean Chem Soc. 2010; https://doi.org/10.5012/bkcs.2010.31.9.2689
